# A qualitative look at bed net access and use in Burkina Faso, Mozambique, Nigeria, and Rwanda following piloted distributions of dual-active ingredient insecticide-treated nets

**DOI:** 10.1186/s12936-024-04868-4

**Published:** 2024-05-07

**Authors:** Jenny Shannon, Moubassira Kagone, Baltazar Candrinho, Sylvanus Otikwu, Chantal Ingabire, Adama Gansane, Samy Pooda, Fofana Aboubacar, Fatou Ouattara, Binete Savaio, Celestino Joanguete, Lucio Sixpence, Hannah Koenker, Perpetua Uhomoibhi, Okefu Oyale Okoko, Onoja Ali, Dele Babarinde, Janet Ogundairo, Ayorinde Samuel Lemah, Aimable Mbituyumuremyi, Joseph H. Singirankabo, Nami Kawakyu, Federica Guglielmo, Christen Fornadel, Kyra Arnett, Joe Wagman, Christelle Gogue, Kenzie Tynuv, Peder Digre, Julia Mwesigwa, Molly Robertson

**Affiliations:** 1 PATH, Seattle, WA USA; 2 PATH, Washington, DC USA; 3 PATH, Kampala, Uganda; 4 PATH, Maputo, Mozambique; 5 Centre National de Recherche et de Formation sur le Paludisme, Ouagadougou, Burkina Faso; 6 National Malaria Control Programme, Ministry of Health, Maputo, Mozambique; 7 Tropical Health LLP, London, UK; 8 National Malaria Elimination Programme, Federal Ministry of Health, Abuja, Nigeria; 9 Ibolda Health International, Abuja, Nigeria; 10 Rwanda Biomedical Centre, Ministry of Health, Kigali, Rwanda; 11 University of Rwanda, Kigali, Rwanda; 12 Liverpool School of Tropical Medicine, Liverpool, UK; 13 IVCC, Liverpool, UK

**Keywords:** Insecticide-treated bed nets, Malaria, Use, Care, Access, Human behavior, Qualitative

## Abstract

**Background:**

Universal coverage with insecticide-treated nets (ITNs) is important for malaria control and elimination. The emergence and intensification of insecticide resistance threatens progress made through the deployment of these interventions and has required the development of newer, more expensive ITN types. Understanding malaria prevention behaviour, including barriers and facilitators to net access and use, can support effective decision-making for the promotion and distribution of ITNs.

**Methods:**

In-depth interviews and focus group discussions were conducted in 3 to 4 villages per district, in 13 districts across Burkina Faso, Mozambique, Nigeria and Rwanda from 2019 to 2022. Interviews were conducted in the local language, translated and transcribed in English, French or Portuguese. Transcripts were coded and analysed using Nvivo and ATLAS.ti.

**Results:**

ITNs were obtained from mass distribution campaigns, antenatal care and immunization visits, and purchased on the private market in some locations. While there were divergent perspectives in whether the number of distributed nets were adequate, participants consistently expressed concerns of bias, discrimination, and a lack of transparency with the distribution process. ITNs were frequently used alongside other malaria prevention methods. The primary motivation for use was malaria prevention. While some participants reported using nets nightly throughout the year, other participants reported seasonal use, both due to the perceived higher density of mosquitoes and discomfort of sleeping under a net in the increased heat. Other barriers to consistent net use included activities that take place away from the home, sleeping patterns and arrangements, and sensitivity to the insecticides on the nets.

**Conclusions:**

ITNs remain an important malaria control intervention. To ensure adequate and increased net access, distribution campaigns should consider family structures, available sleeping spaces, and other bed sharing preferences when identifying the number of nets needed for distribution. In addition, campaigns should allow for multiple options for net distribution points and timing to accommodate households remote to health services. Continuous distribution channels and complimentary distribution through the private sector could help fill gaps in coverage. Solutions are needed for outdoor malaria transmission, including alternative designs for ITNs, and improving access to complementary personal protective measures.

## Background

Optimal coverage with insecticide-treated nets (ITNs) is an essential component of malaria control programmes [1]. ITNs are estimated to have been responsible for 68% of all malaria cases averted in Africa between 2000 and 2015 [2]. Progress toward malaria elimination is threatened by the emergence and intensification of insecticide resistance to pyrethroids, the primary insecticide used for ITNs, in key malaria vector species [3–5]. Accordingly, the need to develop and quickly scale up new malaria vector control tools, including ITNs with novel insecticide formulations, has been identified as a top global public health priority [6]. To meet this challenge, new, dual-active ingredient insecticide-treated nets that use a combination of active ingredients designed to be effective at killing pyrethroid resistant mosquitoes have been developed. The New Nets Project, funded by Unitaid and the Global Fund, was created to increase the market accessibility of dual-active ingredient ITNs to malaria programmes in sub-Saharan Africa.

The project supported the procurement and distribution of a limited quantity of dual-active ingredient ITNs for inclusion in multi-product national distribution campaigns in 2019 and 2020. These distributions were accompanied by observational studies to evaluate the impact and cost-effectiveness of the deployment of these dual-active ingredient ITNs in comparison to standard, pyrethroid-only, ITNs distributed during the same campaigns. The observational studies occurred in five regions across four countries: Burkina Faso, Northern Mozambique, Western Mozambique, Nigeria, and Rwanda. Study districts were selected for inclusion in the study based on (1) which type of ITN was scheduled to be distributed, (2) geographic proximity to one another (for consistency in climate and other geographic features), and (3) baseline comparability in key aspect of malaria transmission (including malaria infection prevalence, malaria case incidence, vector species composition and insecticide-resistance status, and consistencies in other planned malaria control interventions) as described by the most recent Malaria Indicator Surveys, Demographic and Health Surveys (DHS), Reports from PMI and NMPs, and/or relevant peer-reviewed research.

Each study included a qualitative component to characterize malaria prevention behaviour; ITN use, availability and preferences; and perceptions of malaria risk to understand facilitators and barriers to ITN uptake and use. Understanding the sociocultural factors influencing net use and non-use can support interpretation of epidemiological and entomological findings from these observational studies [7, 8]. Improving the understanding of the relationship between the distribution of ITNs and disease prevention can support more effective decision-making in the promotion and distribution of ITNs and more accurate modelling of intervention effectiveness.

## Methods

### Study setting

**Fig. 1:**
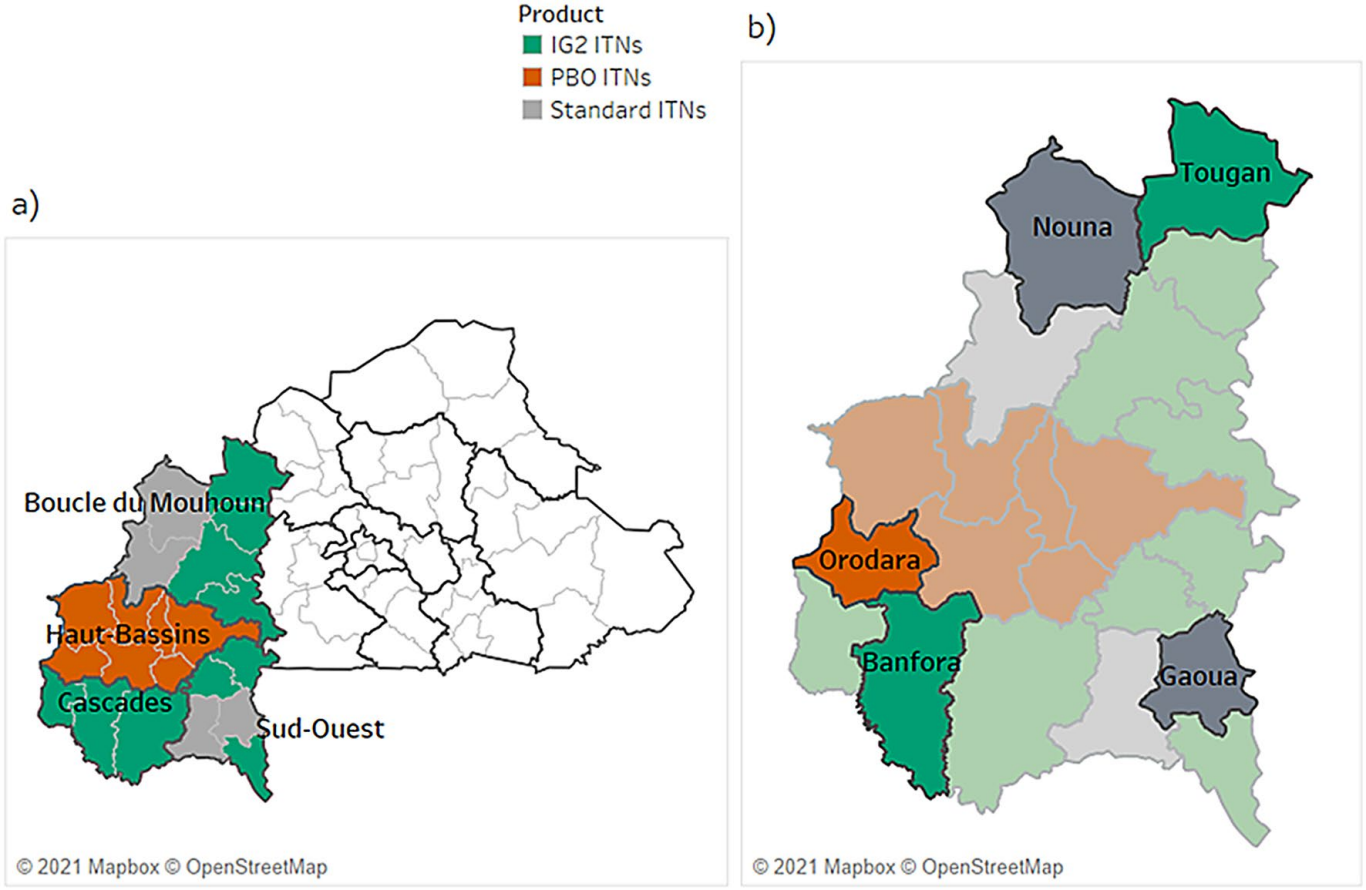
Study districts in Burkina Faso. **a** ITN distribution across four regions of Burkina Faso; **b** the five study districts. *IG2* interceptor G2, *ITN* insecticide-treated bed net, *PBO* piperonyl butoxide

**Fig. 2:**
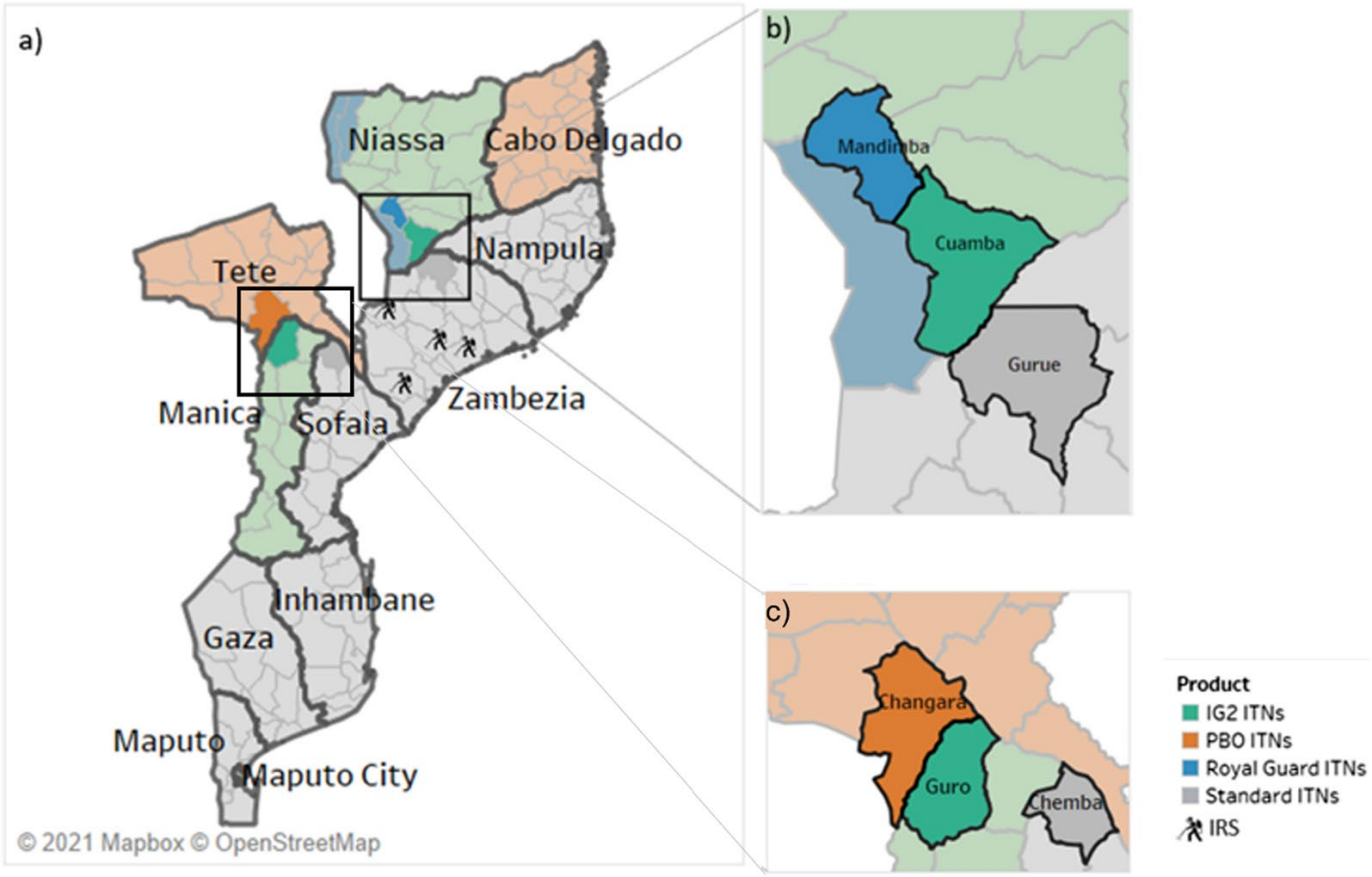
The six study districts in five provinces in northern and western Mozambique. **a** ITN distribution across Mozambique; **b** districts included in the northern evaluation; **c** districts included in the western evaluation. *IG2* interceptor G2, *IRS* indoor residual spraying, *ITN* insecticide-treated bed net, *PBO* piperonyl butoxide

**Fig. 3:**
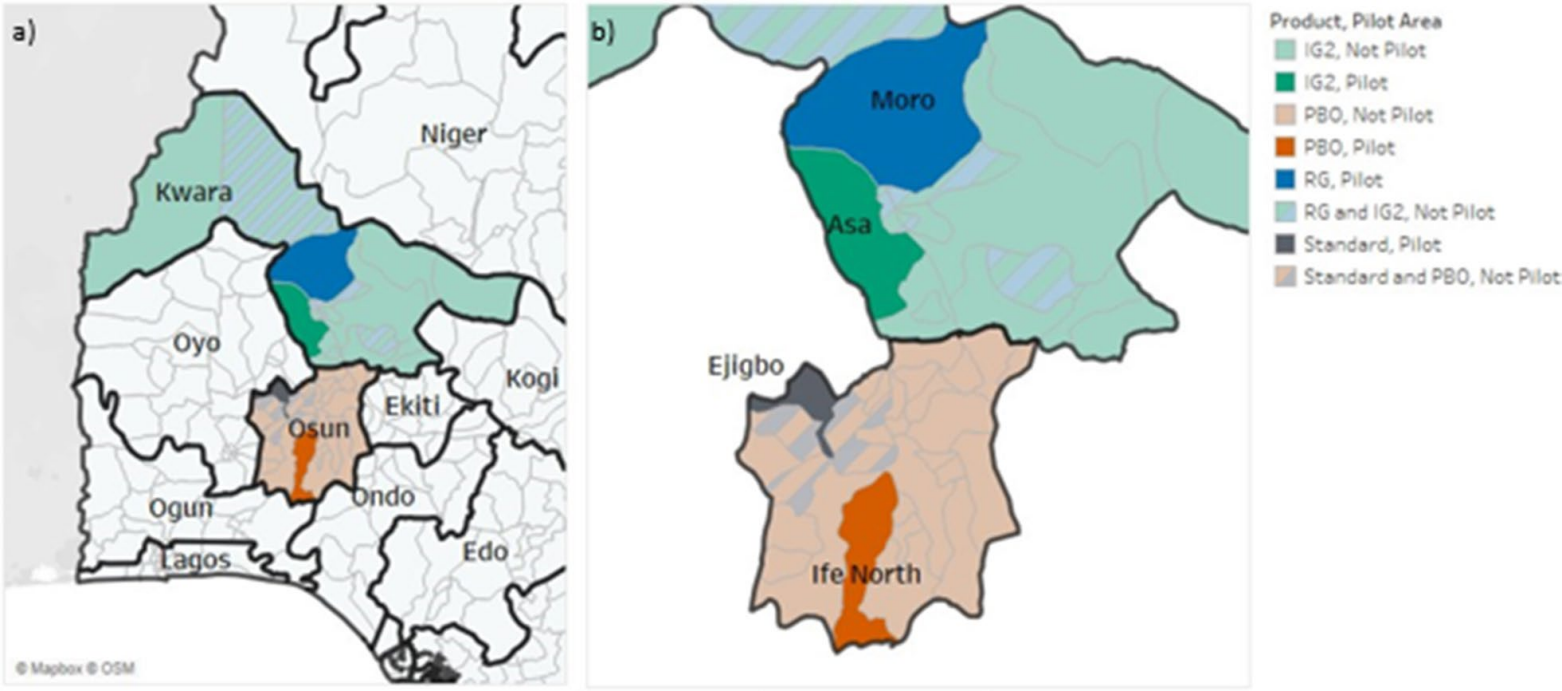
Net distribution in Nigeria by state and local government area. **a** States included in the study; **b** net distribution within Kwara and Osun States, pilot LGAs highlighted. Abbreviations: IG2, Interceptor G2; ITN, insecticide-treated net; PBO, piperonyl butoxide

**Fig. 4:**
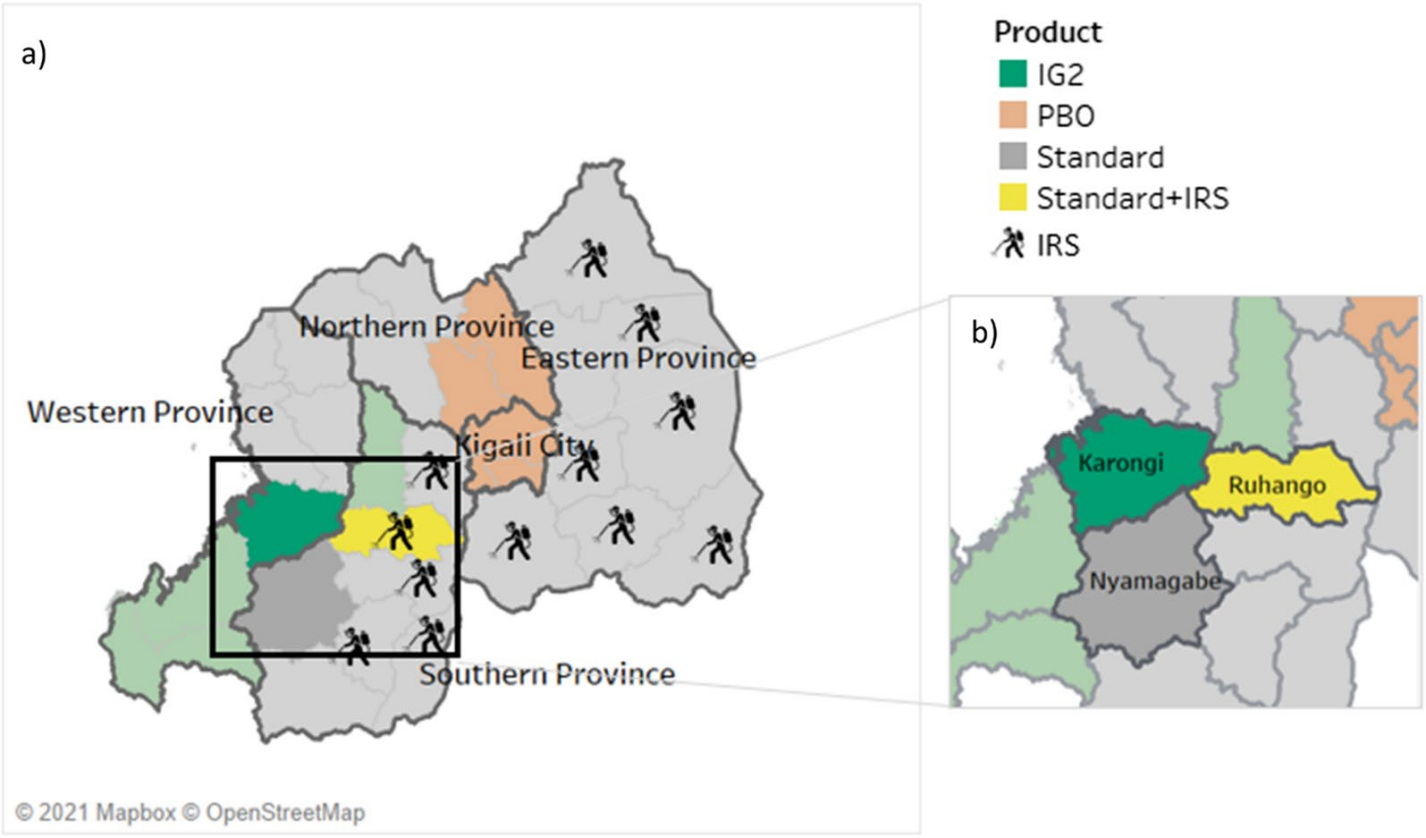
Study districts in Rwanda. **a** ITN distribution across five regions of Rwanda; **b** the three study districts. *IG2* interceptor G2, *IRS* indoor residual spraying, *ITN* insecticide-treated bed net, *PBO* piperonyl butoxide

The national malaria control programmes distributed ITNs in each study district. Qualitative activities occurred in at least three villages in each of the study districts, selected to correspond with entomological surveillance sites (Table 1; Figs. 1, 2, 3, 4). In Mozambique, qualitative activities were carried out in four of six total study districts, selected to include each of the evaluated net types and two districts from each evaluation area (North and West).

**Table 1:** Locations of study sites

Geography	Study districts included	Villages
Burkina Faso	Tougan*, Nouna*, Banfora, Gaoua, Orodara	Panga, Tengrela, Tiefora, Doudou, Holly, Sibera, Dieri, Kourinion, Tin
Northern Mozambique	Cuamba, Mandimba, Gurue*	Lurio Sede, Mepica, Nacaca, Namanha, Camoto, Cuchirimba, Lionde Mitande, Nacolongo
Western Mozambique	Changara, Guro, Chemba*	Cancune, Missaua, Nhalicune, Nhaussua, Cahewe, Gorogode, Nhansana, Tongogara
Nigeria	Asa, Ejigbo, Ife North, Moro	Ajuwon, Alapa, Ballah, Edunabon, Elemere, Ika, Moro, Oke Oyo, Okooko, Ola, Olooru, Shao
Rwanda	Karongi, Nyamagabe, Ruhango	Kizibaziba, Runyinya, Karora, Gitovu, Kigusa, Kivuruga, Gasharu, Karambi, Nyarushishi

^*^No qualitative activities conducted

### Participant selection

Participants for in-depth interviews (IDIs) and focus group discussions (FGDs) were purposively sampled based on observed activities and/or occupation. The inclusion criteria were broad so that all participants who wished to interact with the researchers could be included. A primary criterion for recruitment included physical proximity to the researcher to facilitate multiple interactions throughout the study. Written informed consent was obtained for FGDs and IDIs, which included individuals of both sexes above the age of 18. FGDs were held with groups of 8 to 10 participants. These included heads of households, pregnant women, mothers of children under/over five, men, young adults, and community health workers. Sample sizes were flexible, designed to reach saturation and varied by geography (Table 2).

**Table 2:** Number of FGDs and IDIs in each country

Country	Number of FGDs	Total FGD participants	Number of IDIs	Total IDI participants
Burkina Faso	118	1103	404	404
Mozambique	30	256	–	–
Nigeria	52	415	317	317
Rwanda	96	695	131	131

*FGDs* focus group discussions, *IDI* in-depth interviews

### Data collection

**Fig. 5:**
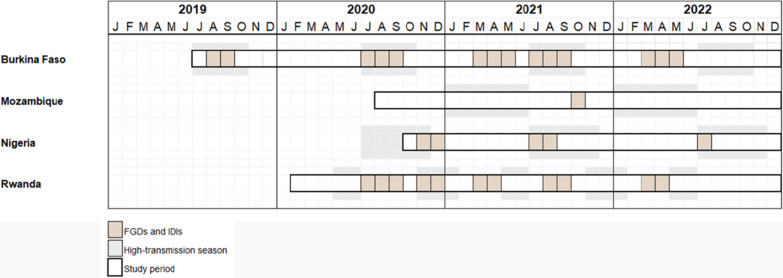
Study timeline by country

Over the four year study, qualitative activities were conducted five times in Burkina Faso and Rwanda, three times in Nigeria and once in Mozambique (Fig. 5). Activities in Mozambique were reduced due to budgetary constraints.

IDIs and FGDs were conducted by research assistants who were trained by qualitative component leads on overall project objectives, ethical considerations, informed consent, COVID-19 mitigation practices, and study methodology and procedures. In each study site, the study team met with local government and representatives from local health facilities, who facilitated introductions and supported activities. Verbal consent was obtained from village chiefs through village sensitization meetings before any study activities were initiated. The researchers provided information on the study objectives and procedures and obtained written consent from participants. Due to the reduced scope of qualitative activities in Mozambique, district health authorities and community health workers identified four villages in each district to conduct activities and facilitated introduction to community leaders.

IDIs and FGDs were conducted using semi-structured guides. They explored malaria experience, strategies for malaria prevention, ITN use, and availability. The IDIs lasted an hour and could occur over multiple sessions if needed. The FGDs occurred in administrative buildings, meeting rooms, health centres, and schools.

### Data management and analysis

The IDIs and FGDs were recorded using digital audio recorders and transcribed. Personal identifying information was removed from transcripts prior to analysis. Transcripts were translated to English prior to analysis in Rwanda and Nigeria. In Burkina Faso and Mozambique, transcripts were analysed in English, French or Portuguese depending on the needs of the researcher. Transcripts were managed and analysed using Nvivo (QSR International) and ATLAS.ti (Scientific Software Development GmbH).

**Fig. 6:**
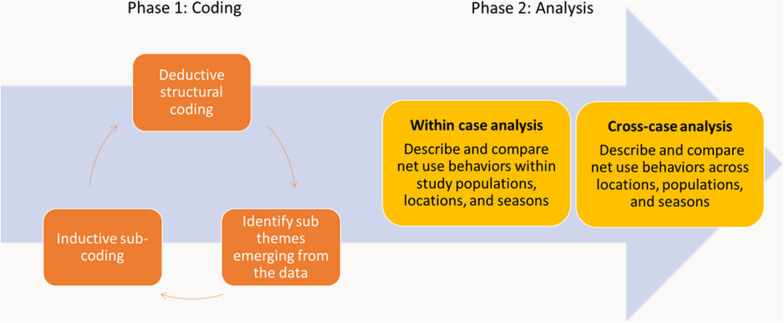
Coding and analysis process

For the primary analysis, data from IDIs and FGDs were analysed thematically [9], concurrent with data collection. Analysts coded a sample of transcripts for interceder reliability. Preliminary coding occurred following initial rounds of data collection to refine semi-structured guides and the codebook. Subsequent rounds of data were coded deductively based on the established codebook, which was validated and revised as needed for each geographic context. A second round of inductive coding identified patterns emerging within deductive codes (Fig. 6). Coding and analysis were conducted by a group of 4 to 5 researchers for each evaluation, including a qualitative lead based in each country along with researchers from the global NNP team involved in all five evaluations.

Secondary analysis was conducted once all data had been collected. Results were compiled by country and theme in an analysis grid, to highlight key findings on ITN availability and use for each country.

## Results

### Net availability

Government mass distribution campaigns were the most common source of ITNs across all study sites. Routine distribution of ITNs during antenatal care (ANC) visits was also a widely reported source, and a few participants reported that they were able to acquire ITNs from community health workers or at a health facility. The retail market for ITNs varied within and across locations, with some participants reporting that ITNs were not sold in their area while others were able to purchase nets to supplement what they receive during distribution. Where nets were not available for purchase, participants felt reliant on government campaigns and were not confident they would be able to replace their nets when they wore out. Where nets were commercially available, many said the cost was too high, the nets were untreated or were perceived as inferior quality compared to the nets given in government campaigns.

*“Before the government programme for supplying free bed nets to all citizens began, bed nets could be sold in many places. But since that programme for free supply of bed net all citizens started, you can’t easily find where you can buy a bed net.”*

FGD, Ruhango, Rwanda

*“My family and I had the mosquito nets during the campaign. When I need more mosquito nets I go to the market and buy, but the nets in the market do not have the same quality with the mosquito nets of the free distribution campaign.”*

FGD, Cuamba, Mozambique

### Government distribution campaigns

Across locations, participants described mass distributions as either door-to-door or based at a central collection point. Similar challenges were heard about the process and frequency of distribution and the number of ITNs distributed to households. For centralized distributions, a household registration was first conducted in the village, where the number of sleeping spaces and/or people per household were recorded. Then, households were given a voucher and instructed to pick up their nets at a central collection point. Participants reported conditions that caused households to be missed and therefore not receive nets, including living in remote areas, moving, traveling or being away at the time of the registration or net distribution, lack of mobility to pick up nets, or losing their ITN voucher after registration.

*“Distribution went well, except for those who lost their census receipts. Those who lost their census receipts did not receive mosquito nets because the distribution agents set conditions. Among the conditions, there is the one that says that "no census receipt, no mosquito net."*

FGD, Gaoua, Burkina Faso

Participants also discussed concerns of bias or a lack of transparency with the distribution process, leading some to believe ITNs were not being distributed fairly. In some cases, the involvement of local authorities was seen to complicate the process or create unfair conditions by involving politics in the process. Others reported discrimination in the distribution process, believing some individuals did not receive ITNs due to marital status, age, or concerns that households were inflating their needs and selling the extra ITNs.

*“I would like the government to distribute mosquito nets in hospitals to avoid the confusion of turning mosquito nets into political campaigns.”*

FGD, Guro, Mozambique

*“During the distribution, there is favoritism, those who distribute can decide to give mosquito nets to people they know well. For example, in some cases we notice that all members of a household receive mosquito nets, but those who do not know anyone distributing the nets are not given the full amount of nets.”*

FGD, Banfora, Burkina Faso

*“Just like my brother has said some do lie and this has reduced trust, therefore this leads to reduction in the number of net given to each household.”*

FGD, Ejigbo, Nigeria

There was divergence regarding whether the number of ITNs distributed were sufficient for all households. Some households, often smaller households with two to three family members, reported that they received the number of ITNs needed for each bed in the house. A smaller number of participants reported receiving extra ITNs that they stored for visitors or as back up for when their nets wear out. It was, however, more common for participants to report that they did not receive an adequate number of nets for their household. Households with insufficient number of ITNs described prioritizing who will use the nets and who will go without, modifying sleeping arrangements to share the nets they have, or continue using old or worn-out nets.

*“Because we were not given enough nets, we had to cut part of our old nets to cover the windows of the rooms where there are not new bed nets.”*

FGD, Ejigbo, Nigeria

*“I have 6 children for two mosquito nets and I don't have a way to protect myself from malaria for all family members, because the net is the only way I have to prevent malaria.”*

FGD, Mandimba, Mozambique

### Net use

Overall, participants from all study sites reported that it is important to use a bed net every night, year-round. However, actual reported net use behaviour varied and ranged from consistent nightly use to occasional or seasonal use, and in rare cases, non-use.

### Facilitators of consistent ITN use

Across all study sites, malaria prevention and/or reducing malaria transmission was the strongest motivating factor for people with access to an ITN to use it nightly. Many participants reported that their use of ITNs increased after personally suffering from a severe case of malaria or witnessing family or neighbors’ experience malaria. Participants observed that cases of malaria in their communities have decreased since the start of the distribution campaigns and credited the use of bed nets with lower instances of malaria in their households and communities.

*“We often used to suffer from malaria before receiving bed nets, and it continued for a while after we received the bed nets because we didn’t know how to use them properly. We later learned to use the bed nets; we learned that we have to fix the bed net every night before we sleep, and fold the bed nets every morning when we wake up. We use the bed net every night even though we close the windows before the nightfall, and remove the bush from the compound.”*

FGD, Ruhango, Rwanda

*“Although before the distribution of mosquito net our children are always down with malaria but when we got to the hospital after testing the child they educate us on the use of mosquito net and ever since then malaria rate has drastically reduced.”*

FGD, Asa, Nigeria

While preventing malaria was the primary driver of net use for most people, participants also noted economic benefits that come along with reduced disease, including saving money on health care expenses and less missed work. Some also reported that nets are less expensive to purchase than other interventions that don’t last as long.

*"Mosquito nets allow us to avoid malaria, mosquito bites as well. In addition, they allow us to avoid expenses for health care. When you sleep under mosquito nets it saves money."*

FGD, Orodara, Burkina Faso

Others reported the added benefit of better sleep, due to the ITN preventing nuisance mosquitos and other insects or pests, protection from dust or debris from the roof, and warmth provided by nets. Some described the peace of mind they felt using a net as a reason they couldn't sleep without one.

*"I use the mosquito net to protect myself against mosquitoes, because they are very effective for us to sleep peacefully at night without mosquito and insect bites, especially scorpions and larvae."*

FGD, Changara, Mozambique

Participants reported seasonal or environmental factors that encourage net use related to perceptions of mosquito abundance and, therefore, perceived malaria threat. Specifically, rain and proximity to bodies of water or forests were reported to influence use.

*“We focus on the rainy season because it favors the mosquito’s reproduction, and malaria prevalence increases compared to the dry season; however, it doesn’t mean that we don’t use the bed net during the dry season.”*

FGD, Karongi, Rwanda

*“Closeness to the river determines the choice of bed net use. Those people living in houses that are far from the river may not use their bed net always, but for us that live close to the river, we always use a bed net.*”

IDI, Asa, Nigeria

### Importance of net use by gender and age

When there were fewer nets than beds in a household, priority was given to pregnant women and young children. Elders were also seen as having a greater risk of malaria, although it was noted by some that older adults may have challenges using nets on their own.

*"Because there are factors of vulnerability to the attack of mosquitoes and other insects, underage children, pregnant women and adolescent girls, these need exclusive nets for many reasons: underage children because they are the easy prey of mosquitoes and contagious diseases of adults; the elderly are also vulnerable to malaria and adolescent girls for reasons of isolation due to hygiene."*

FGD, Changara, Mozambique

*"I know some elders who fail to use the bed net because they cannot manage to fix it properly over their bed frame. Some of them are old and weak to do anything for themselves. They always need someone to assist them with anything."*

IDI, Karongi, Rwanda

Most parents indicated that it is their responsibility to ensure younger children are protected by an ITN by properly securing it on the bed. It is common practice for children to share a net with siblings or parents. It was frequently mentioned that net use is lower among teenagers due to various factors like staying out late in the night, low interest, and lower appreciation of the severity of malaria and/or their susceptibility to it. In some cases in Burkina Faso, it was reported that pregnant women might not use nets due to feeling too hot.

*"As nets are too few, we give priority to children because they are vulnerable and they need help from parents to use the mosquito net correctly, especially the time that must enter the net, care to take into account when leaving and entering the net."*

FGD, Mandimba, Mozambique

*"Some teenagers don’t value the use of a bed net, and we have to push them so that they don’t get sick with malaria. Other teenagers have the knowledge of bed net use and they use it every night to protect themselves against malaria because they love their lives a lot."*

IDI, Karongi, Rwanda

### Use of bed net with other malaria prevention methods

Bed nets were frequently reported to be used along with other malaria prevention methods. Participants in all study sites described the importance of minimizing mosquito breeding grounds by keeping areas around their houses clean and free of brush and grass, eliminating sources of standing water, covering their bodies with clothing, and going indoors and closing doors and windows in the evenings. Other methods included the use of coils, repellent sprays, or burning plants or herbs. Participants often reported that nets are the preferred method, due to ease of use, perceived effectiveness, and cost or side effects of other methods. While bed nets were often reportedly used in conjunction with other methods, in some cases, the use of bed nets eliminated the need for alternative preventative methods used previously that had undesirable results.

*"I use the mosquito net to protect myself from mosquitoes, because they are safe and cheap in relation to eucalyptus leaf fumes that causes coughing and respiratory problems in children when they inhale smoke."*

FGD, Cuamba, Mozambique

*“We clean our environment so that mosquitoes will not have any hiding place around us. We cut the grasses around us and we also make use of mosquito coil and mosquito net. We also close our windows in the evening so that mosquitoes will not enter our house.”*

IDI, Asa, Nigeria

### Barriers to consistent ITN use

Among participants with access to an ITN, some common barriers were reported across all study sites. One of the most frequently cited barriers was activities that keep people away from home, including travel, overnight events like weddings or funerals, or working at night. Night work was mostly associated with men in the study sites, while men and women both participated in other overnight activities. Many of the overnight celebrations mentioned occurred outdoors late into the night or all night. If staying with family or friends, often there were not extra nets for visitors to use. In cases where participants were indoors while away from home, like travel, most said they do not take a net with them, due to nets not being easily portable or because it would leave others in the household without a net. Teenagers were also mentioned as a group that may be out late at night, socializing or attending sporting events.

*"I don't wear mosquito nets at night when I go to talk to my friends in tents or at a death ceremony. The reason for this is that it is difficult to transport mosquito nets to these locations."*

FGD, Changara, Mozambique

*"All months of the year I sleep under the bed net; except when I am not at home, maybe if I go to work in another place far from home, and I have to stay there for some days. In that case, I may not get bed net to use there."*

FGD, Nyamagabe, Rwanda

Other barriers were seasonal; while some participants reported using nets year-round, others prioritized the rainy season, both due to the perceived higher density of mosquitoes and the discomfort of sleeping under a net in the increased heat. Heat was widely reported as a barrier for many participants in all study sites, who either chose not to use a net at all during this time or remove their net for part of the night to cool off. A few participants reported not using their net during the dry season to preserve the net for higher transmission periods.

*"When it is dry season, people think that there are no mosquitoes and don’t use the bed nets every night. However, I think that mosquitoes can come from the wetlands and swamps and bite people and leave them with malaria parasites. People don’t like to use bed net during the dry season because it is hot."*

IDI, Karongi, Rwanda

*"During winter, there is standing water everywhere that promotes the proliferation of mosquitos… therefore the mosquito net is used a lot at this interval. On the other hand, during other times of the year, we have less stagnant dirty water and mosquitoes are less, so others may not use the mosquito net at these times and skip on some days, especially those who have the spirit of protecting their nets for a long time."*

IDI, Orodara, Burkina Faso

Sleeping arrangements also impacted ITN use. When there weren't enough nets for a household, the need to share nets may not align with preferred sleeping arrangements or cultural norms. This was reported especially in Burkina Faso, due to the need for separate sleeping spaces for certain family members, including pregnant women, opposite sex children, particularly those going through puberty, and in polygamous households where men often sleep separately from their wives and children.

*"You know when we take the children there is an age when they reach that they can no longer sleep together if they are of the opposite sex, as from 9 to 10 years old going. So, by grouping them together to give a mosquito net it will not be useful because they cannot sleep together."*

FGD, Banfora, Burkina Faso

*"Sharing [nets] ends up invoking certain myths and taboos of our tradition, for example, no man can sleep in the same net or hammock with his mother-in-law, because it is taboo."*

FGD, Cuamba, Mozambique

Sleeping patterns can change based on season, which can also influence net use. Participants’ reported outdoor net use varied. Many participants across locations said it is not possible to use nets outdoors, while some in Burkina Faso and Mozambique described methods of hanging nets outside when it was too hot to sleep indoors. Some participants in Mozambique suggested that taller, tent shaped nets would be easier to use outdoors.

*"During the heat we attach the mosquito nets outside to sleep. We dig holes and plant wood sticks, then we attach our mosquito net to the end of these sticks. All members of the household who wish to sleep indoors keep their mosquito net indoors and sleep."*

FGD, Banfora, Burkina Faso

Sensitivity to the chemicals used on bed nets was reported as a challenge for many participants and as a reason for not sleeping under a net. This included respiratory issues, skin irritation, and aversion to the smell. However, most participants also reported that this challenge was temporary or could be easily remedied by airing out or washing nets prior to use, and often did not prevent their own use of a bed net. Participants note that proper education on the use of bed nets could prevent this challenge from being a barrier to use for others.

*“Some people do not use mosquito nets because of the chemicals on the net. It has been discovered that some people start using the net immediately when they receive it, against the instruction given to us by the health workers that we should spread it outside for few days before we start using the net. When someone uses the net without spreading it outside, the experience of the adverse effect on their skin may stop them from using the net again.”*

FGD, Asa, Nigeria

### Preferences of net characteristics

Participants evaluated nets based on how easy they are to hang, clean, and use; how well they fit the sleeping space; how they look; and the perceived effectiveness of the insecticide. Preferences of color, shape, and texture varied within and across study sites. These preferences were not mentioned as a facilitator or barrier to use.

Participants who preferred white nets appreciated the clean appearance of the net hanging in their house and the ability to see when the net needs to be washed. Others preferred blue as it doesn’t show dirt as easily. In Nigeria most participants preferred rectangular nets because they better fit the shape of a bed, while participants in Rwanda said that conical nets are easier to hang and take up less space than rectangular nets. In Mozambique, many participants preferred conical nets for indoor use and rectangular nets for outdoor use. Nets with a harder texture were associated with skin irritation, breathing problems, and increased durability. Soft textured nets reportedly kept the user cooler, were easier to wash, and caused less itching. Nets with smaller holes were preferred and seen as more effective than nets with larger holes.

*"I prefer the conical mosquito net because it is easier to fix. Many people don’t like the square shaped mosquito net. Some people have small houses, and when they hang the square shaped mosquito net, it takes up all the space in the house."*

IDI, Karongi, Rwanda

*"There is no type that I do not like; all the bed nets are good, but as for me I prefer the blue colour more than other colours. And the reason is that it doesn’t easily get dirty much like the white one."*

IDI, Ife North, Nigeria

*“I like the soft texture bed net, it feels comfortable sleeping under it. The rough texture of the bed net can cut you.”*

IDI, Karongi, Rwanda

Many participants across study sites preferred longer nets that can easily be tucked in, and some participants requested nets that could be “reboosted” through the application of additional insecticide.

## Discussion

ITNs are one of the most effective tools for malaria prevention and understanding access gaps and patterns of ITN uptake and use are key to guiding decisions and planning for malaria control and elimination strategies. This study explored common factors that influence net use to better understand key barriers to consistent net use.

Even in areas with successful mass distribution campaigns, critical challenges with adequate and equitable distribution of bed nets remained in many communities. In addition to evidence showing a negative correlation between household size and ITN ownership [10, 11], factors such as family structure, available sleeping spaces, and bed sharing practices all indicate that that the standard universal coverage target of one ITN for every two people [12] are not sufficient for many households in some settings. Community concerns and cultural norms around bed sharing must be clearly understood and taken into account to achieve high and equitable household coverage [13]. At a minimum, in communities that struggle to achieve high levels of ITN access and/or use, distribution campaigns should consider target coverages of at least one net per sleeping space [14] to minimize the necessity of bed sharing.

Among those with access to ITNs, key barriers to ITN use still persist. Despite the widespread agreement that bed nets should be used every night throughout the year, seasonal heat continues to be a factor that contributes to inconsistent net use. This is in line with current published research that found discomfort due to warm temperatures to be one of the leading persistent barriers leading to decreased ITN use [15, 16]. Additionally, seasonal variation in use is driven largely by the perception of lower malaria risk during hot, dry seasons. Continued emphasis on the importance of consistent use throughout the year should be included in messaging and education of bed net use, while messaging to encourage increased airing time prior to first use has the potential to improve the frequently mentioned challenge of skin and breathing discomfort due to the insecticide on bed nets.

The use of ITNs outside the home continues to be another challenge. Logistical challenges of carrying a bed net for travel or hanging a net outdoors leave many individuals with little to no protection while away from home. The need for better protection while sleeping outdoors, due to travel, seasonal heat, housing structure, or work responsibilities requires targeted intervention. Improving community understanding of outdoor malaria transmission, making innovative solutions like pop up tents available, and promoting other personal protection measures among those who are outdoors when malaria vectors are active is critical. Providing visual representations of how to use an ITN outdoors, or in other challenging contexts, should also be explored [16].

## Limitations

There are some limitations with this study. One is that the possibility of social desirability bias could result in over reporting net use. This could be especially relevant as a large portion of the qualitative data comes from focus group discussions, where participants could be influenced by the responses of others in the room. However, interviewers and facilitators were trained to emphasize to participants that all experiences of net use, including non-use, were valuable to the study and to create a safe, accepting environment for participants to share their wide-ranging experiences. Along with asking for participants’ own experiences, skilled data collectors asked participants to share reasons that others may not use bed nets as a way to reduce the risk of social desirability bias. Second, the study did not measure the magnitude of the access barriers quantitatively. While this was not the aim, future studies should attempt to quantify some of the barriers identified in this study. Third, these findings are not generalizable outside the study sites. While the findings might be unique to the study contexts, corroboration with existing literature make the data more robust, and lessons drawn from this study can inform the design of interventions elsewhere.

Despite these study limitations, this work contributes to the body of evidence that is foundational to the goal to end malaria and provides relevant context to the quantitative data. The results can inform distribution approaches and social and behaviour change messaging that will help address gaps in bed net access and use.

## Conclusions

Findings from this study identify multiple barriers that must be addressed to improve ITN coverage. Distribution campaigns should consider family structures, available sleeping spaces, and other bed sharing preferences when identifying the number of nets needed for distribution to ensure adequate net coverage within a household. In addition, campaigns should consider multiple options for net distribution points and timing to accommodate households remote to health services. Continuous distribution channels or complimentary distribution of nets through the private sector could help fill gaps in coverage. Other areas of impact include increased communication and behaviour change messaging around the consistent use of ITNs, and exploring solutions for outdoor malaria transmission, including alternative designs for ITNs or similar interventions, and improving access to complementary personal protective measures.

## Data Availability

The datasets used and/or analysed during the current study are available from the corresponding author upon reasonable request.

## Burkina Faso

### General background

Burkina Faso is a 274,200 km^2^ West African Sahelian landlocked country located at a transitional zone between the arid Sahara in the north and the Sudanian zone in the south [17]. The start, duration, and total number of rainy days is therefore highly variable in space and time and defines three ecoclimatic zones: Sahelian zone in the north, Sudanian zone in the south, and Sudano-Sahelian zone in between, with a total annual rainfall and average annual temperature of less than 600 mm/29 °C, 900–1200 mm/28 °C, and 600–900 mm/27 °C, respectively [18]. Nearly 80% of the country’s population work in the agriculture sector and around 70% reside in rural areas [17].

### Local malaria control context

Malaria occurs throughout the year in Burkina Faso, with a peak during the rains between June and October. The 2014 Malaria Indicator Survey estimated malaria prevalence at 45.7% [19]. Several control interventions have been scaled up in a relatively short time in Burkina Faso. The use of artemisinin-based combination therapies, namely artesunate-amodiaquine and artemether-lumefantrine, for uncomplicated malaria was adopted in 2005, and these therapies became available at health facilities in 2007 [20]. Artesunate for severe malaria was adopted in 2012 and made available in 2014 in severe malaria treatment kits at health facilities [21, 22]. Malaria home management by community health workers was pilot tested in 2008 and rolled out countrywide in 2010 [23, 24]. From 2010 to 2013, indoor residual spraying (IRS) was implemented in one health district, Diébougou, and the intervention was halted in 2013. IRS implementation resumed in 2017 in three districts: Kampti, Koungoussi, and Solenzo [25]. Countrywide, insecticide-treated bed net (ITN) mass distribution campaigns were conducted in 2010, 2013, and 2016, with administrative coverage rates of 95.6%, 96%, and 97.41%, respectively [26, 27]. Additionally, population coverage achieved through the mass distribution campaigns has been supplemented by regular distribution of ITNs at all public health facilities through routine antenatal and expanded immunization programmes. Malaria Indicator Surveys, however, showed ITN ownership rates of 90% and 75% in 2014 and 2018, respectively. In 2018, only 33% of households had at least one ITN for two members and only 44% were using their bed nets [28]. Seasonal malaria chemoprevention with sulfadoxine-pyrimethamine plus amodiaquine in the high malaria transmission season has superseded intermittent preventive treatment in children in which the same drug was administered to children on a schedule matching that of the expanded programme on immunization. Lastly, to increase health care seeking at public health facilities, a free of charge health care policy for children under 5 years was implemented in 2016.

### Study sites

A subset of five health districts, Tougan, Nouna, Banfora, Gaoua, and Orodara, were included in the broader study (shown on the map in Fig. 1). These districts had a combined population size of nearly 1.6 million people and 231 health facilities as of 2017. The overall malaria incidence per 1000 people in 2017 was 535 in Nouna, 722 in Gaoua, 370 in Tougan, 729 in Banfora, and 631 in Orodara. The Nouna, Gaoua, Tougan, Banfora, and Orodara health districts are served by 51, 9, 41, 46, and 39 primary health facilities, respectively. They have similar malaria transmission dynamics and consistencies in other malaria control interventions. In addition, their baseline characteristics, determined through routine data provided by in-country stakeholders, were comparable across the districts in underlying malaria prevalence, incidence, vector species composition, and insecticide resistance status, and their climate and geographies are similar.

IG2 ITNs were distributed in Tougan and Banfora; in the two comparator districts, Nouna and Gaoua, standard pyrethroid-only ITNs were distributed. Orodara received piperonyl butoxide (PBO) ITNs. Within each district, ITNs routinely distributed at health facilities were of the same type as those distributed during mass campaigns. Qualitative activities were conducted in three of the five study districts; Banfora, Gaoua, and Orodara.

## Mozambique

### General background

Mozambique is located on the coast of southeastern Africa between South Africa and Tanzania. The country is sparsely populated by 28 million people, and only 36% live in urban areas, including 1 million in the capital, Maputo [29, 30]. There is considerable linguistic diversity in Mozambique: Portuguese is the official language and 26.1% of Mozambicans speak Macua; 8.6% speak Changana; and the rest speak other local languages, of which Lomwe, Sena, and Makhuwa are common in the study districts. The religious makeup of the country is 59.8% Christian, 18.9% Muslim, 4.8% other, and the remaining 16.5% reported either having no religion or did not specify [30]. The communities that make up the study districts are mostly rural, and the chief economic activity is smallholder agriculture, primarily the cultivation of rice, maize, and cassava [31]. In 2017 the literacy rate was estimated to be 60.1% nationally (72.6% for males and 50.3% for females) [32].

### Local malaria control context

Malaria is endemic throughout Mozambique. The country experiences year-round transmission, and risk is heightened during the rainy season, typically from December to April. Malaria cases account for 42% of deaths in children under 5 years and 29% of all deaths overall, yet there are large differences in malaria prevalence and transmission by region. The 2018 combined Survey of Indicators on Immunization, Malaria, and HIV/AIDS showed that prevalence in children under 5 varied throughout the country, ranging from 1% in Maputo Province in the south to 57% in Cabo Delgado Province in the north. Prevalence was generally higher in the northern region (44% to 57%) than in the southern region (1% to 35%), and prevalence in rural areas was more than double that of urban areas (47% compared to 18%). Two provinces, Zambezia and Nampula, represented almost 40% of the national malaria burden. The 2018 indicator survey also showed improvement in ITN coverage compared to the 2011 Demographic and Health Survey: the number of households with at least one ITN increased from 51 to 82%, as did the proportion of children under 5 years and pregnant women reporting having slept under an ITN the night before the survey (36% to 73% and 34% to 77%). However, the number of malaria cases reported increased dramatically from 2012 to 2017, from 3.1 million to more than 8.9 million cases each year, which may be due in part to improved reporting through the routine health management information system [29].

### Study sites

The national malaria control programme prioritized the targeting of dual active ingredient ITNs to two provinces, Manica and Niassa, based on moderate to high malaria infection prevalence rates observed reported in the 2018 combined indicators survey, documented pyrethroid resistance in local vector populations, pre-planned IRS operations targeting the highest-burden districts of Zambezia Province, and the time between net availability and campaign plans. In addition, PBO ITNs were targeted to Cabo Delgado and Tete Provinces based on insecticide resistance patterns. The timing of the planned PBO campaign in Cabo Delgado (July 2019) excluded this province from this study.

Six districts, covering two separate study areas (referred to here as the West and North evaluations) in Niassa and Zambezia Provinces in the north and Manica, Sofala, and Tete Provinces in the west were selected for enhanced study activities to help measure the impact of IG2, Royal Guard® (RG; Disease Control Technologies, LLC), and PBO nets on malaria transmission (see the map in Fig. 2). Qualitative activities were conducted in four of the six study districts; Cuamba, Mandimba, Changara, and Guro.

## Nigeria

### Background

Nigeria is in West Africa, bordered by Niger to the north and Benin and Cameroon to the west and east, respectively. Nigeria is the most populous country in Africa: an estimated 211 million people [33]. The country is divided into 36 states, which are further subdivided into 774 local government areas (LGAs).

### Local malaria control context

Malaria is endemic in Nigeria and a major public health concern, especially for children under 5 and pregnant women. In 2017, there were an estimated 53.7 million malaria cases in the country, representing almost 25% of the entire global burden [34]. Seventy-six percent of the population lives in areas defined by the World Health Organization as at high risk of malaria transmission, while the remaining 24% lives in areas of moderate to low transmission [35].

The primary malaria prevention strategy in Nigeria is the universal distribution of ITNs. The 2018 Demographic and Health Survey indicated that 62% of households owned at least one ITN, and 43% of those surveyed reported having slept under an ITN the night before [36]. In addition, the National Malaria Strategic Plan 2014–2020 includes targeted scale-up of IRS and expanded larval source management as part of an integrated vector management strategy, though IRS is not yet widely implemented [35]. The dominant malaria vector species group in Nigeria (*Anopheles gambiae* s.l.) has been shown to bite readily both indoors and outdoors and has demonstrated moderate to high levels of resistance to pyrethroids, the class of insecticide used on ITNs currently distributed throughout the country.

### Study sites

The four study LGAs are Asa and Moro in Kwara State and Ife North and Ejigbo in Osun State (shown on the map in Fig. 3). The population in these areas ranges from 100,000 to 150,000 people. Study LGAs are in the rain forest or savannah ecological zones and are peri-urban and agricultural. The dominant languages are Yoruba and English, and the most common religions practiced are Islam, Christianity, and traditional religions. Though malaria transmission occurs year-round in Kwara and Osun States, there are seasonal peaks in case incidence and mosquito abundance, typically highest between September and November, during the rainy season. Study activities were conducted in parallel in the pilot study LGAs. Qualitative activities were conducted in all four LGAs.

## Rwanda

### Background

Rwanda is a landlocked country in Central Africa bordered by Burundi, Democratic Republic of the Congo, Tanzania, and Uganda. With 12 million people living within its 26,000 square kilometers, the country is one of the most densely populated in Africa [37]. Over 80% of the population resides in rural areas [38]. The country’s growing service sector provides over 50% of GDP, while the agricultural sector contributes roughly 30%. [37, 39]. Despite a slow annual population growth rate of 1.2% experienced in the 90 s, between 2002 and 2012, the population grew by an average of 2.6% per year [38]. Recently the fertility rate has declined, going from 6.1 in 2005 to 4.2 in 2014 [40]. Kinyarwanda is the primary language, followed by English and French. Kiswahili is also spoken in select areas bordering countries where it is widely spoken. The religious background of the country is 93% Christian, 2% Muslim, and less than 0.5% reporting no religious affiliation [39]. To foster a unified identity and to continue reconciliation efforts after the 1994 genocide, the government introduced a new flag and national anthem in 2001 [41].

### Local malaria control context

Malaria transmission is high throughout the year, but peaks from April to June and from October to December following the two rainy seasons. Approximately 7% of children under 5 and 11% of children 5 to 14 tested positive for malaria by microscopy, according to the 2017 Malaria Indicator Survey. In both cases, prevalence among children in rural areas and children in the lowest wealth quintile was higher than among those in urban areas and those in the highest wealth quintile [42]. From 2005 to 2011, Rwanda significantly reduced its malaria burden, with overall incidence declining 85%, through implementation and scale-up of interventions. From 2012 to 2016, however, malaria incidence increased each year. The largest increases were observed in districts in Southern and Eastern Provinces. The Rwanda Biomedical Centre’s Malaria and Other Parasitic Diseases Division attributed this increase to several factors, including low universal ITN coverage, vector resistance to pyrethroid insecticides, and improvements in health facility reporting and availability of rapid diagnostic tests and artemisinin-based combination therapies. In late 2016 and early 2017, the government of Rwanda distributed more than 5 million ITNs through a mass distribution campaign and implemented IRS with an organophosphate insecticide, expanding coverage from three to five districts. From 2016 to 2017, national incidence stabilized [43]. According to the 2017 Malaria Indicator Survey, 84% of households reported owning at least one ITN, 92% of which were obtained from mass distribution campaigns, 4% from immunization visits, and 2% during antenatal care visits. Sixty-four percent of household populations reported sleeping under an ITN the night before the survey was conducted, including 69% of pregnant women and 68% of children under 5 years [43].

### Study sites

Three districts served as primary study sites for the pilot evaluations: Nyamagabe received standard ITNs, Karongi received IG2 ITNs, and Ruhango received standard ITNs and IRS. Ruhango and Nyamagabe are in Southern Province, and Karongi is in Western Province (see the map in Fig. 4). Baseline characteristics showed comparability across the districts in underlying vector species composition and insecticide resistance status, as well as general climate and geographic similarities. Within each district, ITNs routinely distributed at health facilities were of the same type as those distributed during the mass campaign. Qualitative activities were conducted in all three study districts.

See Figs. 5 and 6.

## Abbreviations

ANC: Antenatal care
FGD: Focus group discussion
IDI: In-depth interview
ITN: Insecticide-treated net
